# Gene bashing of *ceh-6* locus identifies genomic regions important for *ceh-6* rectal cell expression and rescue of its mutant lethality

**DOI:** 10.17912/micropub.biology.000339

**Published:** 2020-12-21

**Authors:** Arnaud Ahier, Shashi Kumar Suman, Sophie Jarriault

**Affiliations:** 1 IGBMC, Development and Stem Cells Department, CNRS UMR7104, INSERM U1258, Université de Strasbourg, Illkirch CU Strasbourg, 67404 France; 2 current address: Clem Jones Centre for Ageing Dementia Research, Queensland Brain Institute, The University of Queensland, Brisbane, Australia

## Abstract

Strong loss-of-function or null mutants can sometimes lead to a penetrant early lethality, impairing the study of these genes’ function. This is the case for the *ceh-6* null mutant, which exhibits 100% penetrant lethality. Here, we describe how we used gene bashing to identify distinct regulatory regions in the *ceh-6* locus. This allowed us to generate a *ceh-6* null strain that is viable and still displays *ceh-6* mutant Y-to-PDA transdifferentiation phenotype. Such strategy can be applied to many other mutants impacting viability.

**Figure 1. Bashing and identification of a cis-regulatory region responsible for the rectal cell-specific ceh-6 expression and rescue of ceh-6 mutants transdifferentiation defects:**
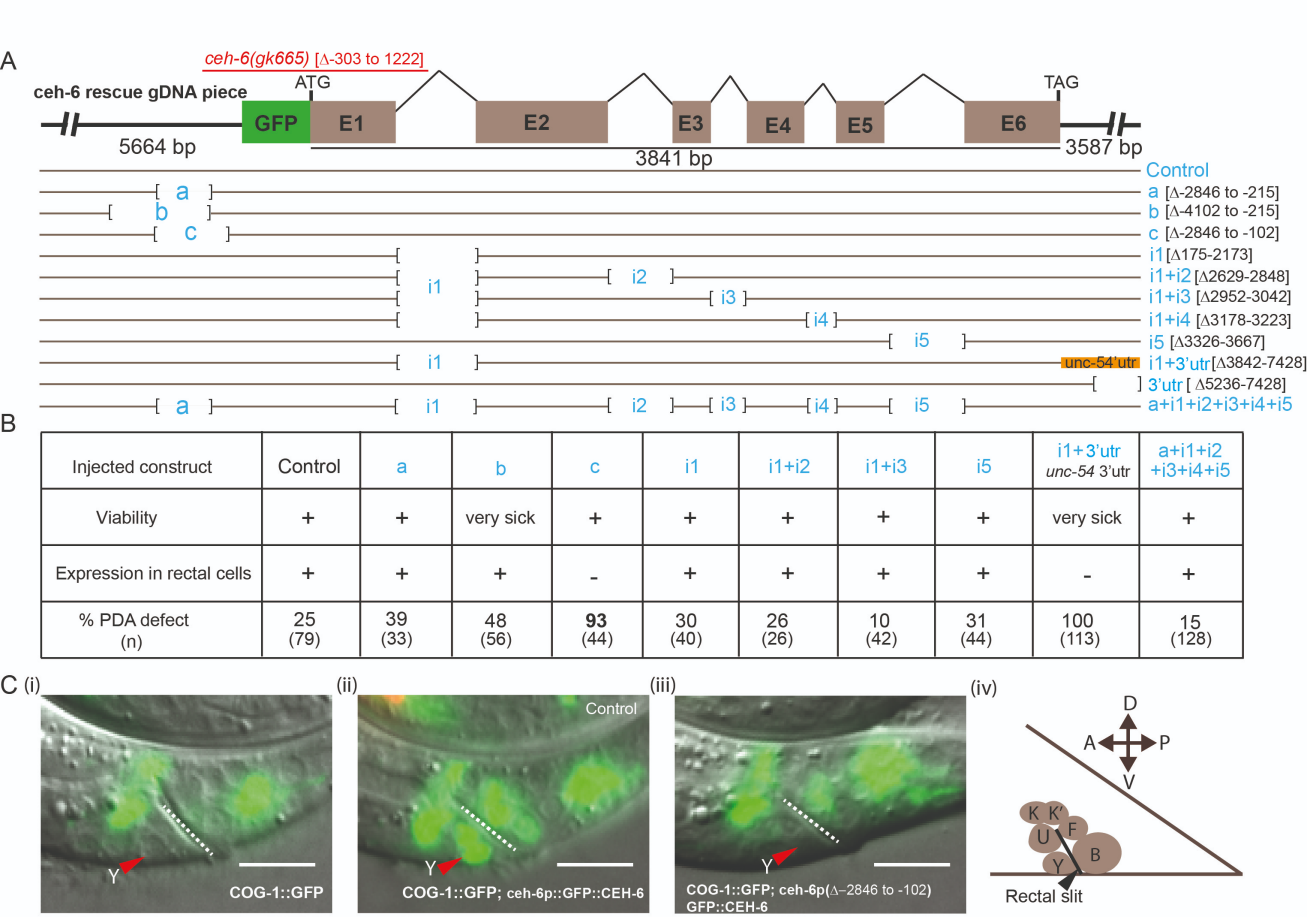
(**A**) Schematic view of the *ceh-6* genomic fragment used for dissection of rectal cell-specific regulatory sequences. The in-frame insertion site of GFP, as well as the location of *ceh-6* ATG and TAG, and each region size are indicated. Deleted regions are represented as closed brackets containing their name in blue (e.g. “a”, “b”, “c”, or the names of the deleted intron i1 to i5 (intron 1-5) and 3’UTR deletion). The location of the deletions with respect to the ATG is also indicated under brackets on the right. Orange: the *ceh-6* 3’UTR was swapped with *unc-54* 3’UTR. (**B**) The ability of the various *ceh-6* constructs to i) rescue *ceh-6(gk665)* lethality; ii) drive expression in rectal cells and iii) rescue *ceh-6(gk665)* defects in transdifferentiation of the Y rectal cell into a PDA neuron is indicated. (n), total number of L3 animals and older scored. Note, constructs ∆3’UTR led to transgenic lines that did not show any expression nor rescue of the lethality, and no lines could be obtained with construct i1+i4. (**C**) Expression pattern of *ceh-6* constructs in the Y and rectal cells. (i), expression pattern of *syIs63* (*cog-1::gfp*) alone for reference; (ii) WT control construct; (iii) “c” construct; (iv), schematic representation of the rectal cells; Red arrowhead, Y cell. Dotted white line, rectal slit.All images were acquired at the L1 larvae stage. Scale bar represents 10 µm.

## Description

Loss of the activity of certain genes, such as *ceh-6*, can lead to lethality at early developmental stages, precluding the study of their function later on during development. Indeed, it was reported that more than 80% of *ceh-6(mg60)* animals died during embryogenesis exhibiting various phenotypes, including an abnormal rectal area and absent excretory canal cell (Bürglin and Ruvkun 2001). *mg60* is a 1.4kb deletion allele that removes *ceh-6* second exon and is believed to cause the null phenotype. The expression pattern of *ceh-6* is complex and matches the reported defects. Most of the *ceh-6* expressing cells, which include head neurons, dividing Pn.a cells in the ventral cord, the excretory cell, and rectal cells, are not related by cell lineage nor by function (Bürglin and Ruvkun 2001).

We have previously shown that knock-down of *ceh-6* activity results in a loss of Y cell transdifferentiation (Td) (Kagias *et al.*. 2012), and that *ceh-6* RNAi inactivation at low dsRNA concentration leads to low penetrance Td defects (Kagias *et al.*. 2012). Since the early lethality associated with strong loss-of-function or null *ceh-6* alleles precludes the study of its role during Td, we sought to engineer a viable *ceh-6* mutant that lacks *ceh-6* activity in the Y cell. One strategy is to drive expression of *ceh-6* in the cells where its activity is needed for viability, but not in the cell where it acts to promote Td. However, the cellular focus for the lethality is unknown, precluding a strategy where expression of *ceh-6* WT cDNA would be specifically targeted to these cells. Since our previous results suggested that *ceh-6* could act cell-autonomously in the Y cell (Kagias *et al.* 2012), we sought to identify the genomic region(s) within the *ceh-6* locus that are necessary for expression in the Y or the rectal cells. Removing these regions from an otherwise *ceh-6* rescuing construct should help to generate a viable *ceh-6* mutant that lacks *ceh-6* expression in the Y or rectal cells. To do so, we have initiated a gene bashing of the *ceh-6* locus and have assessed the ability of the fragments to i) rescue the lethality of *ceh-6* mutants; and ii) still result in Td defect, correlated with a loss of *ceh-6* expression in Y or all rectal cells.

The *ceh-6* gene consists of six exons intervened by five introns, spanning a 3.8kb region (https://wormbase.org/species/c_elegans/gene/WBGene00000431#0-9fb-10). A 13kb fragment for the *ceh-6* locus that includes 5.6kb of upstream sequences and 3.6kb of downstream sequences was used as a template (Fig.1A, control construct). This fragment is able to rescue the phenotypes of *ceh-6(gk665)* mutants, including its lethality and Y Td defect (Fig.1B). In this construct, the GFP sequence was fused in frame with the ATG of the *ceh-6* gene to follow *ceh-6* expression in transgenic animals during rescue experiments (Fig. 1A, C), and we have found it to be expressed in the rectal cells (Fig.1C panel (ii)). A PCR-based approach was used to delete several different regions of the *ceh-6* locus (Fig. 1A). We focused on the first intron of the *ceh-6* gene, by far the largest, as well as on the upstream region as they contain several conserved sequence elements when compared with other *Caenorhabditis* species (ceh-6 UCSC browser). In addition, promoter regions and long first introns have been shown to bear different transcription factor binding sites that may act as additive regulatory regions (Fuxman Bass *et al.* 2013).

Rescue experiments were performed by injecting these constructs in *ceh-6(gk665)* deletion mutants, which bear a 1.5kb deletion encompassing the first exon and most of the first intron (Bürglin and Ruvkun 2001; The *C. elegans* Deletion Mutant Consortium 2012) and exhibit defects similar to the *mg60* allele, including an early lethality (see Methods). No rescue of *ceh-6(gk665)* lethality was obtained when *ceh-6* 3’UTR sequences were altered or when both the first and fourth introns were removed (Fig.1A, constructs ∆3’UTR and I1+I4). In addition, removal of a large upstream region (-4102 to -215, Fig.1A, construct b), or removal of intron 1 plus swapping of *ceh-6* 3’UTR (Fig.1A, constructI1+u-54 3’UTR) led to poor worm survival. These constructs were not further pursued. Constructs with the simultaneous deletion of two or all five introns (Fig.1A) were able to rescue the lethality, suggesting that these regions are dispensable for expression in the cells where lack of *ceh-6* activity causes lethality. However, these constructs still led to expression in the rectal cells and, in large part, rescued the Td defect of *ceh-6* null mutants (Fig. 1B). Large deletions in the upstream region (3887bp and 2631bp resp., Fig.1A, constructs a, b) did not affect expression of the construct in rectal cells either and led to significant rescue of the Td defect. Interestingly, eliminating an additional 113bp closer to the ATG (Fig.1A, construct c) resulted in the complete loss of *ceh-6* expression exclusively in rectal cells (Fig. 1B, Fig. 1C panel (iii)), while its expression appeared normal in other tissues, like the excretory cell and head neurons. Importantly, while construct “c” successfully rescued the lethality of *ceh-6* mutants, transgenic animals exhibited a very penetrant Y Td defect (93%). Thus, most of our constructs rescued *ceh-6(gk665)* lethality. Two constructs, “c” and “i1+3’utr”, resulted in no visible expression in the rectal cells and a corresponding highly penetrant Y-to-PDA transdifferentiation defect, confirming that *ceh-6* activity is necessary in the rectal cells for Y identity swap. Of these two constructs, “c”, which lacks an upstream region, resulted in a relatively healthy transgenic strain.

In summary, we dissected the *ceh-6* gene regulatory sequence in the upstream, intronic and 3’UTR regions. We have identified a small regulatory sequence, located upstream and close to the ATG, that is necessary to drive expression in the rectal cells. A deletion encompassing this region allowed us to build a *ceh-6* synthetic mutant that can be used as a unique tool to study the rectal-specific function of *ceh-6*, for example in Y-to-PDA natural transdifferentiation.

## Methods

All strains were cultured using standard conditions (Brenner 1973). The *ceh-6* genomic loci, encompassed by fosmid WRM0633dB02, was tagged in-frame at the N-terminus with a GFP as described earlier (Tursun *et al.* 2009). To create a *GFP::ceh-6* rescuing construct, GFP-tagged fosmid WRM0633dB02 was used as a template to PCR-amplify a 13993bp fragment using custom-made oligos (table 1), which encompasses the *ceh-6* gene as follows: 5664bp of the *ceh-6* upstream region, *ceh-6* ORF and 3587bp of the downstream sequence. This 13993bp genomic region was cloned into the *pSCB* vector using the *StrataClone* Blunt PCR cloning kit (Agilent Technologies) yielding pSJ6255, which was further used as a parent template to generate all specific deletions as highlighted in Figure 1A. All deletion constructs were made using custom oligos (Table 1) through standard reverse polymerase chain reactions with Phusion^®^ High-Fidelity DNA Polymerase (M0530, NEB) and a Bio-Rad T100 Thermocycler. PCR fragments were phosphorylated using T4 Polynucleotide Kinase (M0201, NEB), and religated using T4 DNA Ligase (M0202, NEB). The *ceh-6* 3’utr was altered by digesting the plasmid with *Sph1* and re-ligation on itself.

To generate *ceh-6* transgenic lines, the *ceh-6* genomic constructs to be tested were injected in IS2581 [*ceh-6(gk665) I / hT2[qIs48]; syIs63[cog-1::gfp; unc-119(+)]*] animals (5ng/µl), together with a co-injection marker *odr-1p::RFP* (pSJ6106, 50ng/µl) and pBSK^+^(200 ng/µl). *hT2[qIs48*] animals are recessive lethal; we found that homozygotes *ceh-6(gk665)* animals are 100% lethal before the L2 stage [as such, all growing progeny from *ceh-6(gk665) / hT2* mother is heterozygote for *ceh-6(gk665)*: 43/43 adults, 33/33 L4 and 53/53 early L3]. Viability was assessed by scoring transgenic *ceh-6(gk665)* adult worms in our transgenic lines. Transgenic *ceh-6* homozygous animals (L3 and older) were scored for the presence of a PDA neuron using the *cog-1p::GFP* marker as previously documented (Richard *et al.* 2011; Zuryn *et al.* 2014).

## Reagents

**Table 1:**

**Table:** 

Plasmid (construct name)	Primer	Strain	Extrachromosomal array	Genotype
pSJ6255	**ceh-6pF** ataagaatGCGGCCGCcgtgttgctttagcacttctccatcccttc **ceh-6 UTR-R** tgatgtgagaagtgaagaggattg	**IS2577**	*fpEx902[GFP::ceh-6 locus 13kb; odr-1::rfp]*	*ceh-6(gk665)I; fpEx902; syIs63[cog-1::gfp;unc-119(+)]IV*
pSJ6321 (a)	**ceh-6gk769extF** ggtggctagacgagacgcagaaag **ceh-6pmidR** gcaacacgccataaataatgaaacc	**IS2639**	*fpEx940[GFP::ceh-6 13kb locus(Δ-2846 to -215); odr-1::rfp]*	*ceh-6(gk665)I; fpEx940; syIs63[cog-1::gfp;unc-119(+)] IV*
pSJ6341 (b)	**ceh-6pmidR2** gccatatcgagtatgaaggatatatc **6gk769extF** ggtggctagacgagacgcagaaag	**IS2691**	*fpEx958[GFP::ceh-6 13kb locus(Δ-4102 to -215); odr-1::rfp]*	*ceh-6(gk665)I/hT2[qIs48]; fpEx958; syIs63[cog-1::gfp;unc-119(+)] IV*
pSJ6342 (c)	**ceh-6pmidR** gcaacacgccataaataatgaaacc **ceh-6PROMmF** cttttgactactacctcttccttttc	**IS2670**	*fpEx955[GFP::ceh-6 13kb locus(Δ-2846 to -102); odr-1::rfp]*	*ceh-6(gk665)I; fpEx955; syIs63[cog-1::gfp;unc-119(+)]IV*
pSJ6317 (i1)	**ceh-6intron1-1f** gtgaactgtaactccagatttttg **ceh-6intron1-1r** ctttatgcctagaaaataacaatctatc	**IS2628**	*fpEx938[GFP::ceh-6 13kb locus(Δ175-2173); odr-1::rfp]*	*ceh-6(gk665)I; fpEx938; syIs63[cog-1::gfp;unc-119(+)]IV*
pSJ6323 (i1+i2)	**ceh-6exon2f** atacacacaagcagatgtaggtg **ceh-6exon2r** cctaatttgattcttctctgctta	**IS2648**	*fpEx945[GFP::ceh-6 13kb locus (Δ175-2173 & Δ2629-2848); odr-1::rfp]*	*ceh-6(gk665)I; fpEx945; syIs63[cog-1::gfp;unc-119(+)] IV*
pSJ6324 (i1+i3)	**ceh-6exon3f** aatatgtgcaaactaaagccac **ceh-6exon3r** cttgaaagagagttgaagcgcttc	**IS2651**	*fpEx948[GFP::ceh-6 13kb locus (Δ175-2173 & Δ2952-3042); odr-1::rfp]*	*ceh-6(gk665)I; fpEx948; syIs63[cog-1::gfp;unc-119(+)] IV*
pSJ6319 (i5)	**ceh-6exon4f** gttgtccgtgtctggttctgcaat **ceh-6exon4r** ctctttctcaagctgcaactccatg	**IS2645**	*fpEx944[GFP::ceh-6 13kb locus (Δ3326-3667); odr-1::rfp]*	*ceh-6(gk665)I; fpEx944; syIs63[cog-1::gfp;unc-119(+)] IV*
pSJ6318 (i1 + 3’utr unc-54 UTR)	**U54swapF** ctcaacagagcccgagacaacaatagcaactgagcgccggtcgctacc **U54swapR** cagcgaccaatgtggaattcgcccttaccgtcatcaccgaaacgcgcgagacg	**IS2603**	*fpEx930[GFP::ceh-6 13kb locus (Δ175-2173 & Δ 3842-7428* + unc-54 3’UTR; odr-1::rfp]	*ceh-6(gk665)I; fpEx930; syIs63[cog-1::gfp;unc-119(+)]IV*
pSJ6355 (a+i1+i2+i3+i4+i5)	**ceh-6gk769extF** ggtggctagacgagacgcagaaag **ceh-6pmidR** gcaacacgccataaataatgaaacc + ligation to cDNA	**IS2624**	*fpEx924[GFP::ceh-6 13kb locus (Δ-2846 to -215 & Δ175-2173 & Δ2629-2848 & Δ2952-3042 & Δ3178-3223 & Δ3326-3667); odr-1::rfp]*	*ceh-6(gk665)I; fpEx924; syIs63[cog-1::gfp;unc-119(+)]IV*
pSJ6325 (i1+i4)	**Ceh-6exon4bf** gtcaatgtaaaatctcgtcttg **Ceh-6exon4br** ctcaatgcttgttctcttctttc	**No lines**		
pSJ6322 (3’utr deletion)	deletion using two *Sph1* natives sites	**Very sick** **No line kept**		
